# Retraction: PARC: a phase I/II study evaluating the safety and activity of pegylated recombinant human arginase BCT-100 in relapsed/refractory cancers of children and young adults

**DOI:** 10.3389/fonc.2026.1993245

**Published:** 2026-09-23

**Authors:** Frontiers Editorial Office

The authors retract the article cited above.

Following publication, the authors contacted the Editorial Office to request retraction of the cited article. Through an internal audit, the sponsor institution found that the laboratory data underpinning the trial could not be independently verified, due to significant concerns regarding sample traceability, storage conditions, analytical procedures, and data reporting. An independent review panel concluded that the robustness of the trial’s results and conclusions was therefore in question. The findings reported in the article are no longer supported by the data; therefore, the article has been retracted.

This retraction was approved the Chief Executive Editor of Frontiers. The authors agree with this retraction.

